# Development of simple predictive models of tooth loss in the community setting

**DOI:** 10.1002/jper.70074

**Published:** 2026-02-28

**Authors:** Michiko Furuta, Takanori Honda, Shinya Kageyama, Nanami Sawada, Satoko Sakata, Jun Hata, Toshiharu Ninomiya, Toru Takeshita

**Affiliations:** ^1^ Section of Preventive and Public Health Dentistry Division of Oral Health, Growth and Development, Faculty of Dental Science Kyushu University Fukuoka Japan; ^2^ Department of Epidemiology and Public Health Graduate School of Medical Sciences Kyushu University Fukuoka Japan; ^3^ Center for Cohort Studies Graduate School of Medical Sciences Kyushu University Fukuoka Japan; ^4^ Department of Medicine and Clinical Science Graduate School of Medical Sciences Kyushu University Fukuoka Japan; ^5^ Department of Health Care Administration and Management Graduate School of Medical Sciences Kyushu University Fukuoka Japan

**Keywords:** community setting, periodontitis, prediction, tooth loss

## Abstract

**Background:**

A simple tool for predicting the risk of tooth loss is considered valuable for the implementation of early preventive measures in community settings. This study aimed to establish a simple predictive model for tooth loss in a community‐dwelling Japanese population.

**Methods:**

This longitudinal study included 1755 participants aged 40–79 years who underwent dental examinations between 2007 and 2012. Incident tooth loss was defined as losing ≥2 teeth over a 5‐year period, corresponding to the highest quartile of the number of teeth lost. Logistic regression analysis was employed for developing multivariate prediction models with and without dental examination data. The developed models were translated into simplified scoring systems based on the beta coefficients. The discrimination of the model was assessed using the Hosmer–Lemeshow test, and the calibration was assessed using a calibration plot.

**Results:**

The incidence of tooth loss was 24.1%. The multivariable model without dental examination data, which included age, current smoking, diabetes, periodontal treatment, number of teeth present, and occupational status as predictors, demonstrated good discrimination and calibration. The multivariable model with dental examination parameters (periodontitis stage and untreated decayed teeth) as predictors demonstrated better predictive performance. The simplified scores, which were developed based on multivariable models, demonstrated predictive performances comparable to that of their respective multivariable models.

**Conclusion:**

The prediction model, even the model without dental examination data can be used to identify individuals at high risk for tooth loss. The simple scoring tool for predicting tooth loss can be readily implemented in the community.

**Plain language summary:**

We developed a multivariable risk prediction model and simplified scoring system for predicting tooth loss, which may help in the identification of individuals at a high risk of tooth loss in a community.

## 1 INTRODUCTION

It has been widely recognized that tooth loss can impact overall health deterioration.^1^, ^2^ Tooth loss leads to challenges in chewing, contributing to dietary restrictions, such as lack of food diversity and inadequate intake of selected nutrients,^3^ resulting in poor nutritional status.^4^ Tooth loss is also associated with functional, esthetic, and social impairments, thereby affecting the individual's quality of life.^5^ Although tooth loss is not a life‐threatening condition, it causes a series of daily limitations. Older individuals with fewer teeth have a shorter and unhealthier life expectancy than those with 20 or more teeth.^6^

Tooth loss remains a significant public health burden, with a recent study reporting that 353 million people globally were edentulous in 2021.^7^ The number of edentulous people globally is expected to increase by nearly 661 million people by 2050, thus representing an annual growth rate of 5.1%.^7^ Tooth loss is highly prevalent but preventable in most cases through proper oral hygiene, regular dental visits, and good health habits.^8^ Therefore, one should focus on early interventions aimed at reducing the risk of tooth loss.

A prediction tool for assessing disease risk can monitor the probability of future outcomes.^9^ Patients with tooth loss reported fear of further tooth loss and complete dysfunction of chewing.^10^ Thus, tooth loss prevention may be a primary concern of such individuals, and awareness of their future risk may motivate behavioral changes toward tooth loss prevention or seeking early professional help.^9^ Hence, a prediction tool would be useful as a preventive intervention for tooth loss at the individual level. This tool could also facilitate targeted prevention strategies for community populations. This prediction tool can aid public healthcare professionals in identifying the future risk of tooth loss in the community. Early identification may promote community‐based intervention programs aimed at reducing tooth loss rates. Therefore, developing an accurate and easy‐to‐use tool for the prediction of future tooth loss in community settings is of great importance.

Although several models have been established for predicting tooth loss,^11^–^15^ some of these models were developed using clinical data from university hospital patients who underwent periodontal treatment.^11^, ^12^, ^13^, ^15^ Thus, these models cannot be applied to individuals without periodontitis and those who have never sought dental care. Although a community‐based study has previously developed a prediction model for tooth loss, this model consisted of self‐reported information and lacked clinical data on the health status and number of teeth present.^14^ Therefore, community‐based cohort studies with clinical examination data are crucial for developing a model that accurately estimates the risk of tooth loss.

This study aimed to establish a prediction model for tooth loss and a simple scoring tool using data from a community‐based cohort study and to evaluate their performance in a community‐dwelling population.

## 2 MATERIALS AND METHODS

### 2.1 Study population

This study was conducted as a part of the Hisayama Study, a community‐based prospective cohort study on cardiovascular disease in the town of Hisayama, a suburb of the Fukuoka metropolitan area in southern Japan.^16^ The demographic characteristics of the study cohort, such as age and occupation, are similar to those of the general Japanese population.^17^ Dental examinations were conducted on Hisayama residents aged ≥40 years in 2007 and 2012 at the health center in Hisayama. Non‐independent older people, including those who required long‐term nursing care, could not participate, and the participation rate was lower for the older age groups. Therefore, participants aged ≥80 years were excluded from this study.^18^

A total of 2665 participants aged 40–79 years underwent both medical and dental examinations in 2007 (70.0% of all residents in this age group).^18^ Similarly, the number of participants was 2325 (57.4%) in 2012. Among the participants in 2007, 1943 underwent dental examinations in 2012 (follow‐up rate: 72.9%). Of these, 94 participants with missing data and 94 participants with 1 or no teeth in 2007 were excluded. Finally, 1755 participants (778 men and 977 women) were examined.

The Kyushu University Institutional Review Board for Clinical Research approved the study protocol (Approval No. 23092‐00). Written informed consent was obtained from all the participants.This study was conducted in accordance with the Helsinki Declaration as revised in 2013.

### 2.2 Tooth loss

The study outcome included the incidence of tooth loss. Tooth loss was evaluated as the difference between the number of teeth present during oral examinations at baseline (2007) and follow‐up (2012). Third molars were excluded from the analysis. This study defined tooth loss as the loss of 2 or more teeth (the highest quartile of the number of teeth lost) over a 5‐year period.

### 2.3 Potential predictors

#### 2.3.1 Assessment of the oral health condition

Untreated decayed teeth were assessed for dental caries. The detailed procedures for periodontal examination in this study have been described elsewhere.^18^ In brief, a periodontal examination was performed according to the Third National Health and Nutrition Examination Survey III (NHANES III) method at 2 sites (mesiobuccal and midbuccal) on all teeth except the third molars using a periodontal probe.* Periodontal status was evaluated using the probing pocket depth (PPD) and clinical attachment level (CAL). The examiners inserted the probe parallel and close to the tooth to minimize interference from calculus or restorations. When the cemento‐enamel junction was obscured, its position was estimated from adjacent teeth or normal anatomy. In 2007, the inter‐examiner agreement for PPD and CAL measurements within ± 1 mm between 8 examiners and the gold‐standard examiner was very high (kappa > 0.8).^18^ Periodontitis was defined based on the classification proposed at the 2018 World Workshop^19^ as interdental clinical attachment loss at ≥2 non‐adjacent teeth or buccal/oral clinical attachment loss of ≥3 mm with a pocket depth of > 3 mm in ≥ 2 teeth. Considering the staging of periodontitis, clinical attachment losses of 1–2 mm, 3–4 mm, and ≥5 mm were defined as stage I, II, and III, respectively. By assessing maximum PPD (stage I, PPD ≤4 mm; stage II, PPD ≤5 mm; stage III, PPD ≥6 mm), stage shifting was possible only when the stage increased. For example, stage II would shift to stage III if the maximum PPD was ≥6 mm. As this study did not examine the reasons for the missing teeth, the number of teeth present as a stage‐shifting complexity factor was used to discriminate between stages III and IV; fewer than 20 teeth were classified as stage IV.^20^ Periodontitis in the 2018 classification is categorized as follows: no, stage I to II, and stage III to IV. In addition to the primary definition of periodontitis based on the 2018 classification, having ≥1 tooth with PPD ≥4 mm was evaluated as an alternative indicator of periodontal condition.

#### 2.3.2 Clinical and biochemical assessments

The plasma glucose levels, systolic and diastolic blood pressures, and body mass index (BMI) were measured. A 75‐g oral glucose tolerance test was conducted for all participants, except for those with severe diabetes or those undergoing insulin treatment. Diabetes was defined as either undergoing treatment for diabetes with medication and/or insulin injections, or a fasting plasma glucose ≥126 mg/dL, 2 h post‐prandial, and random plasma glucose ≥200 mg/dL, or both.^21^ Hypertension was defined as systolic blood pressure ≥140 mmHg, diastolic blood pressure ≥90 mmHg, or ongoing antihypertensive treatment.^22^ Obesity was defined as BMI ≥ 25.0 kg/m^2^, which was the optimal cutoff for obesity in Asian individuals.^23^

#### 2.3.3 Questionnaire

A self‐administered paper questionnaire was mailed to the participants before the examination. Completed forms were collected upon arrival at the health center. All participants returned the questionnaire. A self‐administered questionnaire was used to assess toothbrushing frequency, regular dental visits, periodontal treatment, smoking habits, and occupational status. The frequency of tooth brushing was categorized as once per day or less and twice per day or more. Dental visits were categorized as regular visits to oral care at least once a year. Periodontal treatment was dichotomized as periodontal treatment within 1 year/over 1 year ago versus no/do not know. Smoking status was dichotomized as current smoker versus former/never smoker. Occupational status was classified into 3 categories: clerical support workers; homemakers, unemployed, or retired; and other jobs. Other jobs included professional or technical occupations (skilled workers), sales, service, agriculture, forestry, fishery, and manual labor.

### 2.4 Statistical analysis

This study considered 2 models for developing the prediction model: a model without dental examination data and a clinical examination model. A model without dental examination data can be used when clinical information, such as periodontal conditions and dental caries, is not available. This model considered the following variables at baseline as predictors: age (40–49, 50–59, 60–69, 70–79 years), sex (men, women), number of teeth present (≥28 teeth, 20–27 teeth, ≤19 teeth), regular dental visit (yes, no), periodontal treatment (no, yes), current smoking (no, yes), diabetes (no, yes), obesity (no, yes), hypertension (no, yes), occupational status (homemakers, unemployed, or retired; clerical support workers; and other jobs). Although this study built the model without dental examination data, including the number of teeth present, diabetes, obesity, and hypertension from clinical information, we anticipate that information on these variables can be obtained from the questionnaire. The number of teeth present as self‐reported in the questionnaire was validated.^24^

The prediction model was constructed using a logistic regression model with backward variable selection, in which the criterion for variable elimination was set at *p* < 0.20. For backward variable selection, the first model included the abovementioned variables in the model without dental examination data, and these variables included the periodontitis stage (no, stage I–II, III–IV) and untreated decayed teeth (no, yes) in the clinical examination model. Another clinical examination model included having at least 1 tooth with PPD ≥ 4 mm (no, yes) instead of the periodontitis stage.

Once the final models were determined, a simplified scoring system was developed based on the beta coefficients of these multivariable logistic regression models, followed by the method used in the Framingham Heart Study.^25^ We calculated the number of points assigned to each variable by dividing the beta coefficient by the smallest coefficient in the model and rounding this quotient to the nearest integer. The predicted 5‐year probability was determined using the following formula: *p*^ = 1/[1+exp{‐(Intercept + smallest coefficient × total score)}].

The agreement between the 5‐year probability of tooth loss predicted by the multivariable model and that predicted by the simplified score was assessed using Spearman's rank correlation and bivariate linear regression of the model‐predicted probability based on the score‐predicted probability.

The discrimination of the multivariable model and simplified score was assessed using C‐statistics. For internal validation, a 200‐cycle bootstrap process was performed to assess the optimism of the developed model, and the optimism‐corrected C index was calculated according to the procedure proposed by Lankham and Slaughter.^26^ Calibration was assessed graphically by plotting the average predicted 5‐year probabilities against the observed 5‐year probabilities using the locally estimated scatterplot smoothing (Loess) function. The Hosmer–Lemeshow (HL) *χ* ^2^ test was performed for quantitatively assessing the calibration.

The sensitivity analysis examined multivariable models with and without dental examination data, and tooth loss was defined as the loss of ≥1 or ≥3 teeth.

A 2‐tailed *p*‐value < 0.05 was considered statistically significant (except for backward elimination). Statistical software† was used for all statistical analyses.

## 3 RESULTS

The participants’ characteristics are listed in Table 1. The mean number of teeth present was 24.1 (standard deviation, 5.8). The percentage of participants with untreated decayed teeth was 28.6%. The percentage of participants was 34.5% and 30.9% in stages I–II and III–IV of periodontitis, respectively.

**TABLE 1:** Baseline characteristics of the study participants

Variables	Mean (standard deviation) or frequency
Age, years	59.5 (9.9)
40–49, %	17.8
50–59, %	32.1
60–69, %	31.2
70–79, %	18.9
Women, %	55.7
No. of present teeth	24.1 (5.8)
≥28, %	23.2
20‐27, %	60.8
≤19, %	16.0
Presence of untreated decayed teeth	28.6
Periodontitis stage	
No periodontitis	34.5
Periodontitis stage I/II	34.5
Periodontitis stage III/IV	30.9
Presence of PPD ≥4 mm	48.7
≤1 time/day of toothbrushing frequency, %	31.8
No regular dental visit, %	72.4
Periodontal treatment, %	30.5
Current smoking, %	19.4
Diabetes, %	14.8
Obesity, %	27.3
Hypertension, %	42.9
Occupational status	
Homemaker, unemployed, or retired, %	49.7
Clerical support workers, %	25.4
Other jobs, %	24.9

Abbreviation: PPD, probing pocket depth.

During the 5‐year follow‐up period, 24.1% of the participants experienced tooth loss. The univariate association between potential predictors and the risk of tooth loss is shown in Table S1 in the online *Journal of Periodontology*.

### 3.1 Model without dental examination data

In the model without dental examination data, multivariable analysis with backward variable selection revealed that age, current smoking, diabetes, periodontal treatment, number of teeth present, and occupational status were significant predictors of incident tooth loss (Table 2).

**TABLE 2:** Multivariable model for predicting tooth loss: Model without dental examination data

Variables	(Reference)	*β* coefficient	SE	Odds ratio (95% CI)	*p*‐value
Age groups (years)					
50–59	(vs. 40–49 years)	0.43	0.22	1.54 (0.99–2.39)	0.055
60–69		0.82	0.23	2.27 (1.46–3.55)	<0.001
70–79		1.23	0.25	3.42 (2.12–5.54)	<0.001
Current smoking		0.38	0.15	1.46 (1.08–1.98)	0.013
Diabetes		0.26	0.16	1.29 (0.95–1.76)	0.105
Periodontal treatment		0.52	0.13	1.69 (1.32–2.15)	<0.001
Number of present teeth					
20–27 teeth	(vs. ≥28 teeth)	1.42	0.23	4.14 (2.66–6.45)	<0.001
≤19 teeth		2.23	0.26	9.30 (5.64–15.34)	<0.001
Occupational status					
Clerical support workers	(vs. homemaker, unemployed, or retired)	0.27	0.17	1.31 (0.94–1.84)	0.111
Other jobs		0.31	0.15	1.36 (1.02–1.82)	0.036
Index of the predictive ability for the multivariable model					
C‐statistics	0.743 (0.727–0.769)				
Optimism‐corrected C‐statistics	0.734				

*Note*: Only the variables selected by backward elimination are presented. The optimism‐corrected C‐statistics were calculated based on 200 bootstrap samples.

Abbreviations: CI, confidence interval; SE, standard error.

**FIGURE 1:**
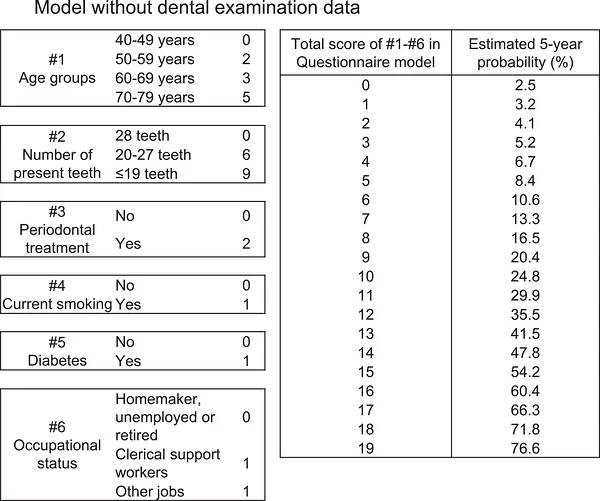
Simplified point‐based scoring system for tooth loss in the model without dental examination data. After adding points #1 to #6, the predicted 5‐year risk of tooth loss can be determined. The probabilities are shown on the right.

Based on the final multivariable prediction model, the calculation of the simplified score and the corresponding predicted 5‐year probability of tooth loss are presented in Figure 1. The total score ranged from 0 to 19. The estimated 5‐year probability according to the total score is shown in Figure 1. The predicted 5‐year probabilities of tooth loss by the multivariable model and the simplified score were highly linearly correlated (Spearman's correlation coefficient *r* = 0.996; intercept = −0.004; and regression coefficient *β* = 0.982 [95% confidence interval [CI] 0.977 to 0.986] in a bivariate linear regression model that regressed the model‐predicted probability on the score‐predicted probability). The C‐statistic for the multivariate model was 0.743 (95% CI, 0.727–0.769) in the original cohort. The optimism‐corrected C‐statistic was 0.734 using 200 bootstrap samples drawn randomly with replacements from the original cohort.

**FIGURE 2:**
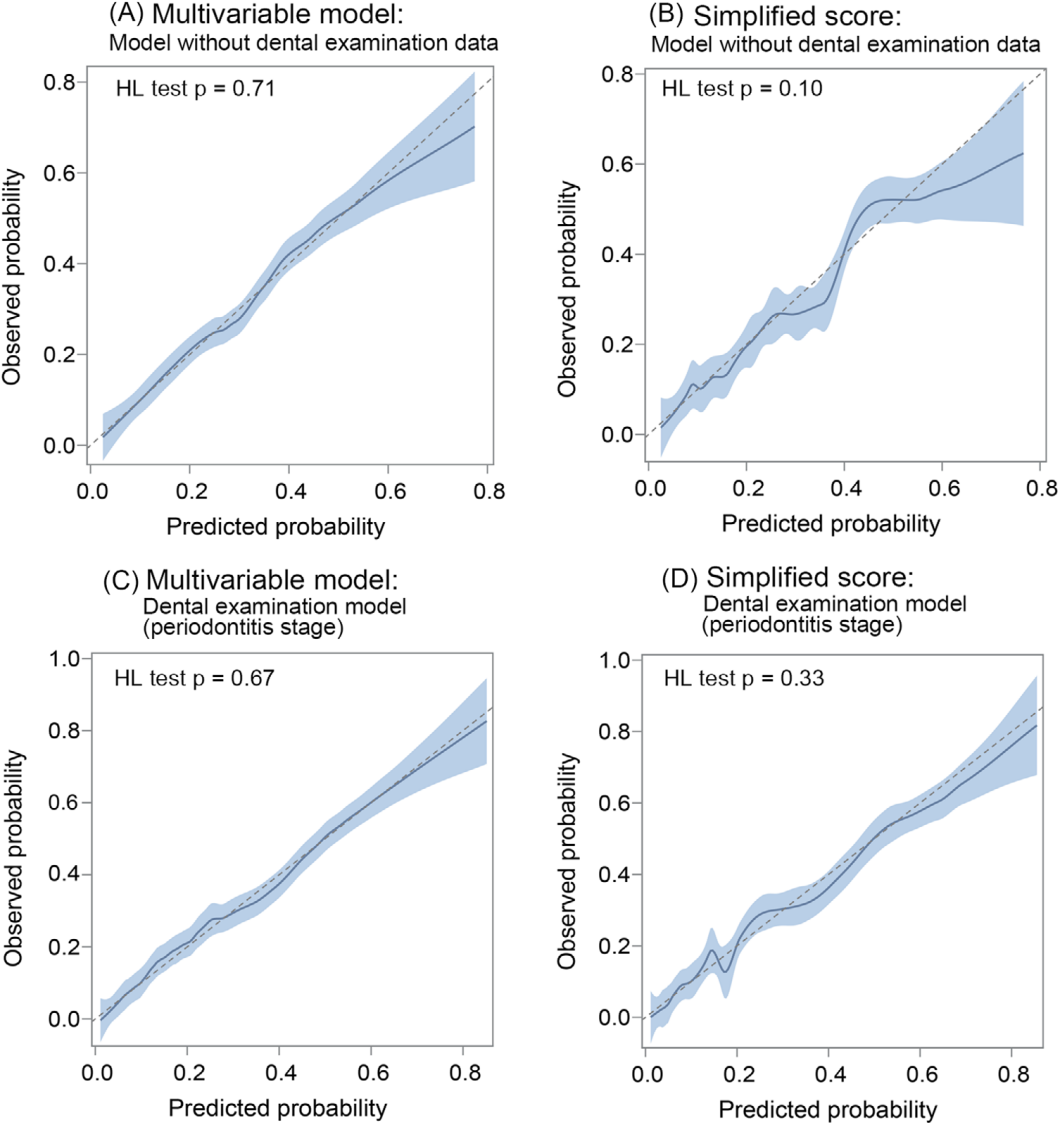
Calibration plot of the observed probability against the model estimated probability in the model without dental examination data: (A) multivariable model and (B) simplified score, and the clinical examination model including the periodontitis stage: (C) multivariable model and (D) simplified score. Loess regression is a nonparametric method that employs locally weighted regression to create a smooth curve through data points in a scatter plot. The shaded region indicates the 95% confidence interval for the Loess curve. HL test, the Hosmer–Lemeshow test.

Calibration plots demonstrated that the model‐predicted 5‐year probability and observed probability were highly linearly correlated (Figure 2a), and HL tests indicated good calibration of the model (*p* = 0.71). The predicted 5‐year probability according to the simplified score also demonstrated a linear correlation with the observed probability (Figure 2b), with a *p*‐value of 0.10 for the HL test.

### 3.2 Clinical examination model

**FIGURE 3:**
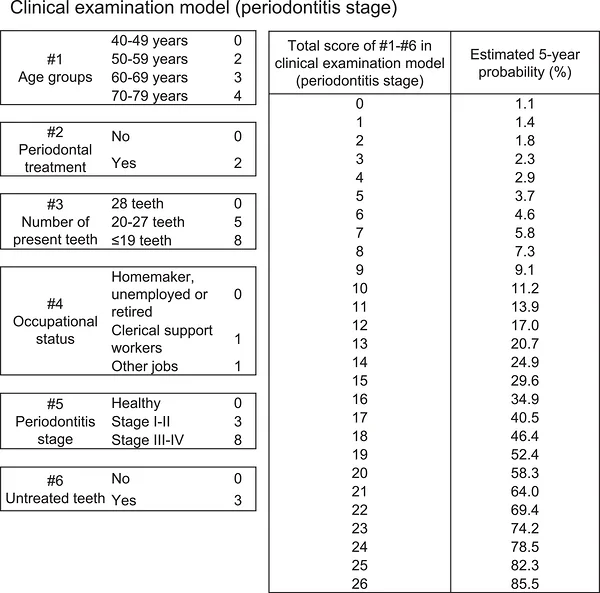
Simplified point‐based scoring system for tooth loss in the clinical examination model, including the periodontitis stage.

In the clinical examination model, age, periodontal treatment, number of teeth present, occupational status, periodontitis stage, and the presence of untreated decayed teeth were selected as significant predictors of tooth loss (Table 3). The simplified score and predicted 5‐year probability of tooth loss are shown in Figure 3. The predicted 5‐year probabilities of tooth loss by the multivariable model and the simplified score were highly linearly correlated (Spearman's correlation coefficient *r* = 0.997; intercept = 0.004; and regression coefficient *β* = 0.967 [95% CI 0.963–0.971]). The C‐statistic for the multivariate model was 0.806 (95% CI, 0.784–0.829) in the original cohort, and the optimism‐corrected C‐statistic was 0.801. The calibration plots demonstrated good agreement with observed probabilities (Figures 2c,d), and the HL tests showed good calibration (*p* = 0.67 and 0.33). The results were similar when periodontitis was defined as ≥1 tooth with PPD ≥4 mm (Table S2 and Figure S1 in the online *Journal of Periodontology*).

**TABLE 3:** Multivariable model for predicting tooth loss with dental examination data: Clinical examination model (periodontitis stage)

Variables	(Reference)	*β* coefficient	SE	Odds ratio (95% CI)	*p*‐value
Age groups					
50–59	(vs. 40–49 years)	0.37	0.24	1.45 (0.91–2.31)	0.117
60–69		0.61	0.24	1.84 (1.15–2.94)	0.011
70–79		1.05	0.26	2.87 (1.73–4.74)	<0.001
Periodontal treatment		0.41	0.13	1.50 (1.16–1.95)	0.002
No. of present teeth					
20–27	(vs. ≥28 teeth)	1.30	0.23	3.66 (2.32–5.79)	<0.001
≤19		1.93	0.26	6.90 (4.11–11.57)	<0.001
Occupational status					
Clerical support workers	(vs. homemaker, unemployed, or retired)	0.28	0.18	1.31 (0.94–1.84)	0.122
Other jobs		0.24	0.15	1.36 (1.02–1.82)	0.118
Periodontitis stage					
Stages I–II	(vs. Healthy)	0.76	0.20	2.14 (1.46–3.14)	<0.001
Stages III–IV		1.87	0.19	6.49 (4.48–9.38)	<0.001
Presence of untreated decayed teeth		0.68	0.13	1.97 (1.51–2.56)	<0.001
Index of the predictive ability for the multivariable model					
C‐statistics	0.806 (0.784–0.829)				
Optimism‐corrected C‐statistics	0.801				

*Note*: Only the variables selected by backward elimination are presented. The optimism‐corrected C‐statistics were calculated based on 200 bootstrap samples.

Abbreviations: CI, confidence interval; SE, standard error.

### 3.3 Different definitions of tooth loss

In the model without dental examination data, the C‐statistic was 0.727 (0.703–0.750) for ≥1 tooth lost and increased to 0.744 (0.712–0.776) for ≥3 teeth lost, similar to the value for ≥2 teeth lost (Tables S3 and S4).

In the clinical examination model, the corresponding C‐statistics were 0.766 (0.744–0.788) for ≥1 and 0.812 (0.786–0.839) for ≥3 teeth lost (Tables S4 and S5). Calibration plots showed a strong linear relationship and good agreement between predicted and observed probabilities (Figures S2 and S3).

## 4 DISCUSSION

The present study developed a multivariable risk prediction model and simplified scoring system to predict tooth loss over 5 years based on a community‐based cohort study. The proposed prediction models demonstrated good discrimination, acceptable calibration, and high internal validity. These models employed predictive factors that are commonly measured during periodic community health checkups. Therefore, the simplified score is readily implementable in the community and may help identify individuals who may benefit from early intervention aimed at reducing the risk of tooth loss.

This study proposed 2 models: a model without dental examination data and a clinical examination model including dental caries and periodontitis. A model without dental examination data can be used when oral examination data are unavailable. The variables for each model were selected using a logistic regression model with backward variable selection. Both models included age, periodontal treatment, number of teeth present, and occupational status. Dental caries and periodontitis remained in the final clinical examination model, whereas smoking and diabetes did not, likely because their effects were captured by clinically assessed periodontitis. In contrast, the model without dental examination data included current smoking and diabetes instead of dental caries and periodontitis. Accumulated evidence has indicated that smoking^27^ and diabetes^28^ are associated with periodontitis. Smoking and diabetes may serve as alternative risk factors for periodontitis in the model without dental examination data. Periodontal treatment was selected as the predictor in both models as it may reflect a history of periodontitis. Thus, individuals receiving such treatment may be more susceptible to recurrent disease and, subsequently, tooth loss.

Recently, several prediction models for tooth loss have been developed using machine learning algorithms and deep learning models.^11^, ^12^, ^13^, ^14^ The prediction models using machine learning were fitted to learn the conditional distribution of the outcome based on a set of predictors without strict assumptions on the data distributions, nonlinear associations, and interactions.^29^ Machine learning methods are capable of powerful predictions by detecting complex relationships in the data. Several machine‐learning models for predicting molar loss demonstrated superior performance compared with logistic regression models.^12^ Despite these advantages, the contributions of individual predictors can be hard to interpret in complex predictive models using machine‐learning algorithms.^30^ Many machine learning models have issues that impose certain challenges in initiating interventions for reducing the risk of poor health outcomes because the models do not directly demonstrate how each feature influences the outcome.^31^ Conversely, tree‐based machine learning systems, such as RuleFit, are easily interpreted.^32^ Additionally, this algorithm allows the use of continuous or counted data, as well as follow‐up time, which contributes to improving predictive accuracy by accounting for the temporal influence on tooth loss.^32^ Although our prediction models provide explicit and interpretable estimates of predictor effects, future studies are needed to confirm whether the machine learning systems can further enhance predictive performance.

The simplified scoring system allows the calculation of the predicted probability of tooth loss using a list of scores for the items and the estimated probability according to the total score. As shown in the example of a simplified score when the questionnaire information is available (Table S5), the predicted probabilities of tooth loss can be easily calculated. Our simplified scoring system is intuitive, making it easier for users to quickly understand or interpret the results.

In Japan, municipalities conduct dental checkups for residents aged 20–70 years in 10‐year intervals, which is termed “Periodontal Disease Screening” under the Health Promotion Act.^33^ The periodontal examination uses the Community Periodontal Index (CPI). The CPI code ≥ 1, or having at least 1 tooth with PPD ≥ 4 mm, is considered as suspected of periodontitis.^33^ To enhance community applicability, we also used an alternative definition of at least 1 tooth with PPD ≥4 mm. Models incorporating this criterion showed good predictive ability (Table S2), suggesting that tooth loss can be predicted even without a detailed periodontal examination.

We developed prediction models using alternative definitions of tooth loss (≥1 and ≥ 3 teeth lost). Predictors such as age, number of teeth present, periodontal treatment, periodontitis stage, and untreated teeth were consistently identified across all definitions, with minor differences in other factors. All models demonstrated acceptable predictive performance. Meanwhile, in our participants, losing at ≥1 tooth was relatively frequent (44.0%); the corresponding model showed a lower C‐statistic than the model for ≥2 teeth lost, suggesting that ≥1 tooth lost may be less suitable for risk stratification. Furthermore, ≥3 teeth lost was less frequent (13.8%) and may represent a more severe outcome. Therefore, ≥2 teeth lost appears to be an appropriate balance for identifying individuals at higher risk who may benefit from early preventive measures.

Our prediction models were developed using predictors commonly available in community settings. As dental examinations are not part of Japan's annual health checkups, a model without dental examination data can be readily incorporated into routine screenings. Such tools may raise individual awareness about oral conditions and encourage healthier behaviors. Future studies should examine whether tooth loss screening based on our model improves oral health awareness and behavior.

Tooth loss increases with age. A national survey in Japan in 2022 reported that the mean number of teeth present was 27.9, 26.4, 24.8, 21.0, and 15.6 in individuals aged 40–44, 50–54, 60–64, 70–74, and 80–84 years, respectively.^34^ Therefore, older adults may lose several teeth within a decade. Thus, 5‐year prediction models may be more suitable than long‐term models for guiding early prevention and intervention.

The reasons for tooth extraction (e.g., dental caries or periodontitis) were not investigated owing to the limited examination time. A Japanese nationwide survey showed that dental caries and periodontitis contribute similarly to tooth extraction at 40–59 years of age, whereas periodontitis becomes dominant at 60–79 years of age.^35^ Older individuals in our study may have lost more teeth due to periodontitis than dental caries. Our prediction model, without dental examination data, included periodontitis‐related factors such as diabetes, smoking, and periodontal treatment. Examination of the reasons for tooth extraction may result in a different model of tooth loss due to dental caries. Combining all reasons for tooth extraction may influence predictive performance. Future studies should examine the causes of tooth loss to improve predictor selection and provide more targeted interventions. However, examining the overall incidence of tooth loss remains clinically meaningful, because it represents the cumulative burden of oral diseases and reflects the outcomes that individuals ultimately experience.

Our study has some potential limitations. First, we did not conduct an external validation of the proposed model owing to data unavailability. Therefore, further validation across diverse populations is warranted. Second, our prediction model included self‐reported variables such as current smoking and periodontal treatment. While participants could accurately indicate their current smoking status, some may have forgotten or misunderstood whether they had received periodontal treatment. Participants who were unsure about their experiences with periodontal treatment were categorized as having no treatment. Although recall bias or misclassification might occur in periodontal treatment, the β coefficient in the multivariable prediction model and the corresponding point in the simplified scoring system were relatively small in periodontal treatment, suggesting a limited influence on the model's overall accuracy. This limited impact may be partly because other variables, such as the number of teeth and periodontitis, played a dominant role in the prediction model. Third, our study population did not include individuals requiring long‐term care or those with 1 or no teeth at baseline. This selection may limit the applicability of our models to dependent older adults with a higher burden of oral disease. However, we focused on dentate community‐dwelling individuals to develop models to identify potentially preventable incident tooth loss for which early preventive measures can be realistically implemented. Fourth, the 2007 survey did not collect data on the use of dental floss or interdental brushes, preventing us from evaluating their effects on tooth loss. Fifth, a partial‐mouth assessment was used to evaluate periodontal conditions, omitting the examination of lingual or palatal sites, which may have resulted in an underestimation of the actual periodontal status. Although this underestimation could attenuate the predictive strength of periodontitis stage or lead to the misclassification of individuals with more severe undiagnosed disease, full‐mouth partial diagnostic records in the 2018 classification of periodontitis were found to be effective in estimating the prevalence and severity.^36^ Periodontitis stage remained a predictor in our clinical examination model, suggesting that its contribution to risk stratification was retained. Nonetheless, future studies incorporating full‐mouth examinations are warranted to confirm the generalizability of our findings. Sixth, due to the lack of data on radiographic bone loss or CAL measured 5 years prior to baseline, we were unable to assess the grade of periodontitis according to the 2018 classification system. Finally, this study was conducted among residents of a single municipality in Japan. Owing to the small sample size, the generalizability of our findings to a broader Japanese population is limited, and caution should be exercised when applying these results to other populations.

## 5 CONCLUSIONS

In conclusion, we developed risk prediction models and a simplified scoring system for tooth loss for use in community settings. Prediction tools could improve individuals’ understanding of their accumulated risk and facilitate the early identification of those at high risk of tooth loss in a community.

## AUTHOR CONTRIBUTIONS

*Conceived and designed the study*: Michiko Furuta, Takanori Honda, Toshiharu Ninomiya, and Toru Takeshita. *Conducted statistical analysis*: Michiko Furuta and Takanori Honda. *Data collection*: Michiko Furuta, Takanori Honda, Shinya Kageyama, Satoko Sakata, Jun Hata, Toshiharu Ninomiya, and Toru Takeshita. *Interpreted the data and drafted the* *article*: Michiko Furuta, Takanori Honda, Shinya Kageyama, Nanami Sawada, Toshiharu Ninomiya, and Toru Takeshita. All authors critically reviewed and approved the final manuscript.

## CONFLICT OF INTEREST STATEMENT

The authors declare no conflicts of interest relevant to this study.

## Supporting information



Supporting Information

## Data Availability

The data are not publicly available due to ethical restrictions.

## Footnotes

* PCP11, Hu‐Friedy, Chicago, IL, USA.

† The Statistical Analysis System software package, version 9.4, SAS Institute, Cary, NC, USA.
