# Supplementary material for: Development of simple predictive models of tooth loss in the community setting
Source: J Periodontol. 2026 Feb 28;97(8):1795–806. doi: 10.1002/jper.70074 (PMC13502424; doi:10.1002/jper.70074)
Supplement: Supplementary file 1 [file JPER-97-1795-s001.docx]

SUPPLEMENTARY MATERIALS

**Development of simple predictive models of tooth loss in the community setting**

**Authors:** Michiko Furuta, Takanori Honda, Shinya Kageyama, Nanami Sawada, Satoko Sakata, Jun Hata, Toshiharu Ninomiya, Toru Takeshita

| Supplementary Table 1. Unadjusted association of potential predictors with the risk of tooth loss | | | |
| --- | --- | --- | --- |
| Variables | % of tooth loss | Crude OR | (95%CI) |
| Age |  |  |  |
| 40-49 years | 10.2 | 1 |  |
| 50-59 years | 18.3 | 1.96 | (1.28-3.00) |
| 60-69 years | 27.6 | 3.35 | (2.22-5.05) |
| 70-79 years | 41.4 | 6.20 | (4.05-9.49) |
| Gender |  |  |  |
| Men | 27.8 | 1 |  |
| Women | 21.2 | 0.70 | (0.56-0.87) |
| Number of present teeth |  |  |  |
| ≥28 teeth | 5.9 | 1 |  |
| 20-27 teeth | 24.5 | 5.18 | (3.35-8.00) |
| ≤19 teeth | 49.3 | 15.54 | (9.67-24.97) |
| Untreated teeth |  |  |  |
| No | 20.1 | 1 |  |
| Yes | 34.1 | 2.05 | (1.63-2.58) |
| Periodontitis stage |  |  |  |
| No periodontitis | 7.3 | 1 |  |
| Periodontitis stage I/II | 20.6 | 3.32 | (2.31-4.78) |
| Periodontitis stage III/IV | 46.8 | 11.22 | (7.91-15.93) |
| Presence of PPD ≥4 mm |  |  |  |
| No | 10.9 | 1 |  |
| Yes | 38.1 | 5.03 | (3.92-6.47) |
| Toothbrushing frequency |  |  |  |
| ≥2 times/day | 25.6 | 1 |  |
| ≤1 time/day | 23.4 | 0.89 | (0.70-1.12) |
| Regular dental visit |  |  |  |
| Yes | 25.2 | 1 |  |
| No | 23.7 | 0.92 | (0.72-1.17) |
| Periodontal treatment |  |  |  |
| No | 19.5 | 1 |  |
| Yes | 34.5 | 2.17 | (1.73-2.73) |
| Current smoking |  |  |  |
| No | 23.2 | 1 |  |
| Yes | 27.9 | 1.29 | (0.98-1.68) |
| Diabetes |  |  |  |
| No | 22.3 | 1 |  |
| Yes | 34.8 | 1.86 | (1.40-2.47) |
| Obesity |  |  |  |
| No | 23.4 | 1 |  |
| Yes | 26.1 | 1.16 | (0.91-1.48) |
| Hypertension |  |  |  |
| No | 20.8 | 1 |  |
| Yes | 28.9 | 1.53 | (1.23-1.90) |
| Job |  |  |  |
| Homemaker, unemployed or retired | 24.4 | 1 |  |
| Clerical support workers | 19.3 | 0.74 | (0.56-0.98) |
| Other jobs | 28.4 | 1.23 | (0.95-1.59) |
| CI, confidence interval; PPD, probing pocket depth. | |  |  |

| Supplementary Table 2. Multivariable model for predicting tooth loss with dental examination data: Clinical examination model (PPD ≥4 mm) | | | | | |  |
| --- | --- | --- | --- | --- | --- | --- |
| Variables | (reference) | β coefficient | SE | Odds ratio (95%CI) | p value | |
| Age groups |  |  |  |  |  | |
| 50–59 years | (vs 40–49 years) | 0.48 | 0.23 | 1.61 (1.02-2.54) | 0.041 | |
| 60–69 years |  | 0.75 | 0.23 | 2.12 (1.34-3.35) | 0.001 | |
| 70–79 years |  | 1.19 | 0.25 | 3.28 (2.00-5.37) | <0.001 | |
| Periodontal treatment |  | 0.48 | 0.13 | 1.62 (1.26-2.10) | <0.001 | |
| Number of present teeth |  |  |  |  |  | |
| 20–27 teeth | (vs ≥28 teeth) | 1.28 | 0.23 | 3.60 (2.29-5.67) | <0.001 | |
| ≤19 teeth |  | 2.05 | 0.26 | 7.78 (4.66-12.98) | <0.001 | |
| Job |  |  |  |  |  | |
| Clerical support workers | (vs Homemaker, unemployed or retired) | 0.32 | 0.18 | 1.38 (0.98-1.96) | 0.067 | |
| Other jobs |  | 0.29 | 0.15 | 1.34 (1.00-1.80) | 0.054 | |
| Presence of ≥1 tooth with PPD ≥4 mm | | 1.30 | 0.15 | 3.69 (2.83-4.81) | <0.001 | |
| Presence of untreated teeth |  | 0.68 | 0.13 | 1.98 (1.52-2.57) | <0.001 | |
| Index of the predictive ability for the multivariable model | |  |  |  |  | |
| C-statistics | 0.796 (0.773-0.819) |  |  |  |  | |
| Optimism-corrected C-statistics | 0.790 |  |  |  |  | |
| PPD, probing pocket depth; SE, standard error; CI, confidence interval. | | | | | |  |
| Only the variables selected by backward elimination are presented. | | | | | |  |
| The optimism-corrected C-statistics was calculated based on 200 bootstrap samples. | | | | |  | |

| Supplementary Table 3. Multivariable models for predicting **the loss of ≥1 tooth** | | | | |  |
| --- | --- | --- | --- | --- | --- |
| Variables | (reference) | β coefficient | SE | Odds ratio (95%CI) | p value |
| **Model without dental examination model** | | | | |  |
| Age groups |  |  |  |  |  |
| 50-59 years | (vs 40-49 years) | 0.43 | 0.17 | 1.54 (1.11-2.14) | 0.009 |
| 60-69 years |  | 0.78 | 0.17 | 2.18 (1.57-3.03) | <0.001 |
| 70-79 years |  | 1.07 | 0.19 | 2.90 (2.00-4.22) | <0.001 |
| Men |  | 0.27 | 0.11 | 1.31 (1.07-1.61) | 0.011 |
| Diabetes |  | 0.30 | 0.15 | 1.35 (1.01-1.81) | 0.044 |
| Periodontal treatment | | 0.42 | 0.11 | 1.53 (1.22-1.91) | <0.001 |
| Number of present teeth | |  |  |  |  |
| 20-27 teeth | (vs ≥28 teeth) | 1.15 | 0.15 | 3.17 (2.38-4.22) | <0.001 |
| ≤19 teeth |  | 1.99 | 0.20 | 7.31 (4.97-10.75) | <0.001 |
| Index of the predictive ability for the multivariable model | | | | |  |
| C statistics | 0.727 (0.703-0.750) |  |  |  |  |
| Optimism-corrected C statistics | 0.722 |  |  |  |  |
|  |  |  |  |  |  |
| **Clinical examination model (periodontitis stage)** | | | | |  |
| Age groups |  |  |  |  |  |
| 50-59 years | (vs 40-49 years) | 0.39 | 0.20 | 1.47 (1.05-2.07) | 0.026 |
| 60-69 years |  | 0.62 | 0.17 | 1.87 (1.32-2.64) | <0.001 |
| 70-79 years |  | 0.92 | 0.18 | 2.50 (1.69-3.71) | <0.001 |
| Regular dental visit |  | 0.28 | 0.13 | 1.33 (1.04-1.70) | 0.024 |
| Diabetes |  | 0.23 | 0.15 | 1.26 (0.93-1.70) | 0.138 |
| Periodontal treatment |  | 0.26 | 0.12 | 1.29 (1.02-1.64) | 0.035 |
| Number of present teeth | |  |  |  |  |
| 20-27 teeth | (vs ≥28 teeth) | 1.03 | 0.15 | 2.81 (2.09-3.77) | <0.001 |
| ≤19 teeth |  | 1.72 | 0.20 | 5.56 (3.73-8.28) | <0.001 |
| Periodontitis stage |  |  |  |  |  |
| Stage I-II | (vs Healthy) | 0.52 | 0.14 | 1.68 (1.29-2.19) | <0.001 |
| Stage III-IV |  | 1.31 | 0.14 | 3.70 (2.79-4.91) | <0.001 |
| Presence of untreated teeth | | 0.51 | 0.12 | 1.66 (1.31-2.10) | <0.001 |
| Index of the predictive ability for the multivariable model | | | | |  |
| C statistics | 0.766 (0.744-0.788) |  |  |  |  |
| Optimism-corrected C statistics | 0.761 |  |  |  |  |
| SE, standard error; CI, confidence interval. | |  |  |  |  |
| Only the variables selected by backward elimination are presented. | | | | | |
| The optimism-corrected C statistics was calculated based on 200 bootstrap samples. | | | | |  |

| Supplementary Table 4. Multivariable models for predicting **the loss of ≥3 teeth** | | | | |  |
| --- | --- | --- | --- | --- | --- |
| Variables | (reference) | β coefficient | SE | Odds ratio (95%CI) | p value |
| **Model without dental examination model** | |  |  |  |  |
| Age groups |  |  |  |  |  |
| 50-59 years | (vs 40-49 years) | 0.74 | 0.32 | 2.09 (1.14-3.85) | 0.018 |
| 60-69 years |  | 0.92 | 0.31 | 2.51 (1.37-4.60) | 0.003 |
| 70-79 years |  | 1.03 | 0.33 | 2.79 (1.47-5.30) | 0.002 |
| Current smoking |  | 0.28 | 0.19 | 1.32 (0.91-1.93) | 0.149 |
| Men |  | 0.43 | 0.16 | 1.54 (1.13-2.10) | 0.006 |
| Periodontal treatment |  | 0.36 | 0.15 | 1.44 (1.07-1.93) | 0.0162 |
| Number of present teeth | |  |  |  |  |
| 20-27 teeth | (vs ≥28 teeth) | 1.38 | 0.31 | 3.99 (2.17-7.34) | <0.001 |
| ≤19 teeth |  | 2.56 | 0.33 | 12.94 (6.73-24.88) | <0.001 |
| Index of the predictive ability for the multivariable model | | | | |  |
| C statistics | 0.744 (0.712-0.776) |  |  |  |  |
| Optimism-corrected C statistics | 0.736 |  |  |  |  |
|  |  |  |  |  |  |
| **Clinical examination model (periodontitis stage)** | | | | |  |
| Age groups |  |  |  |  |  |
| 50-59 years | (vs 40-49 years) | 0.65 | 0.32 | 1.91 (1.02-3.60) | 0.045 |
| 60-69 years |  | 0.67 | 0.32 | 1.95 (1.04-3.64) | 0.036 |
| 70-79 years |  | 0.81 | 0.33 | 2.25 (1.17-4.40) | 0.015 |
| Periodontal treatment |  | 0.22 | 0.16 | 1.25 (0.92-1.71) | 0.156 |
| Number of present teeth | |  |  |  |  |
| 20-27 teeth | (vs ≥28 teeth) | 1.19 | 0.32 | 3.28 (1.76-6.10) | <0.001 |
| ≤19 teeth |  | 2.15 | 0.34 | 8.56 (4.40-16.65) | <0.001 |
| Periodontitis stage |  |  |  |  |  |
| Stage I-II | (vs Healthy) | 0.68 | 0.28 | 1.97 (1.15-3.38) | 0.014 |
| Stage III-IV |  | 2.00 | 0.26 | 7.41 (4.49-12.22) | <0.001 |
| Presence of untreated teeth | | 0.49 | 0.16 | 1.63 (1.19-2.23) | 0.002 |
| Index of the predictive ability for the multivariable model | | | | |  |
| C statistics | 0.812 (0.786-0.839) |  |  |  |  |
| Optimism-corrected C statistics | 0.805 |  |  |  |  |
| SE, standard error; CI, confidence interval. | |  |  |  |  |
| Only the variables selected by backward elimination are presented. | | | | | |
| The optimism-corrected C statistics was calculated based on 200 bootstrap samples. | | | | |  |

| Supplementary Table 5. Clinical examples of 5-year tooth loss risk prediction using the simplified score in hypothetical subjects. | | |
| --- | --- | --- |
| Clinical characteristics | Example A | Example B |
| Age | 55 | 68 |
| Current smoking | Yes | No |
| Diabetes | No | Yes |
| Periodontal treatment | No | Yes |
| Number of present teeth | 24 teeth | 19 teeth |
| Job | Skilled worker | Retired |
|  |  |  |
| Simplified score, points | 10 | 15 |
| Score-predicted 5-year tooth loss risk | 25% | 54% |
| (A) an individual aged 55 years is a current smoker and skilled worker with 24 teeth; and (B) an individual aged 68 years has diabetes, experienced periodontal treatment, and 19 teeth.  Based on the simplified score for each item and the estimated 5-year probability of tooth loss in the total score (Figure 1), the predicted probabilities of tooth loss for (A) and (B) were calculated as 25% and 54%, respectively. | | |

**Supplementary Figure 1.** Simplified point-based scoring system for tooth loss in the clinical examination model including the presence of ≥1 tooth with PPD ≥4 mm (a). Calibration plot of the observed probability against the model estimated probability: (b) multivariable model and (c) simplified score. PPD, probing pocket depth.

**Supplementary Figure 2.** Simplified point-based scoring system for the **loss of ≥1 tooth** in (a) the model without dental examination data and (b) the dental examination model (periodontitis stage). Calibration plots comparing the observed and model-estimated probabilities in the model without dental examination data: (c) multivariable model and (d) simplified score.　Calibration plots comparing the observed and model-estimated probabilities in the dental examination model (periodontitis stage): (e) multivariable model and (f) simplified score.

**Supplementary Figure 3.** Simplified point-based scoring system for **loss of ≥3 teeth** in (a) the model without dental examination data and (b) the dental examination model (periodontitis stage). Calibration plots comparing the observed and model-estimated probabilities in the model without dental examination data: (c) multivariable model and (d) simplified score.　Calibration plots comparing the observed and model-estimated probabilities in the dental examination model (periodontitis stage): (e) multivariable model and (f) simplified score.
